# Comparison of TLC and Different Micro TLC Techniques in Analysis of Tropane Alkaloids and Their Derivatives Mixture from *Datura Inoxia* Mill. Extract

**DOI:** 10.1007/s10337-013-2414-x

**Published:** 2013-03-15

**Authors:** Irena Malinowska, Marek Studziński, Karolina Niezabitowska, Maria Gadzikowska

**Affiliations:** 1 Department of Planar Chromatography, Faculty of Chemistry, Maria Curie-Skłodowska University, Lublin, Poland; 2 Department of Inorganic Chemistry, Pharmaceutical Faculty with Medical Analysis Division, Medical University of Lublin, Lublin, Poland

**Keywords:** TLC, HPTLC, UTLC, ETLC, Tropane alkaloids

## Abstract

Planar chromatography is a very useful tool for analysis of wide range of different mixtures. Thanks to its possibility for rapid separation of large number of samples simultaneously, low solvent consumption and ability to analyse rough material allow to receive precise and reliable results in short time and low cost. Miniaturization of planar techniques brings a lot of advantages, such as shortening distance and time of chromatogram development, and further lowering of solvent consumption. Besides, it often allows to improve separation parameters and raise efficiency of chromatographic system. In this paper, ability of analysis of tropane alkaloids mixture from *Datura Inoxia* Mill. extract using conventional TLC technique with five micro TLC techniques (short distance TLC, HPTLC, UTLC, OPLC and ETLC) in maximally closed chromatographic conditions was compared in order to present abilities of micro TLC techniques in plant material analysis.

## Introduction

Applying a micro TLC technique or adapting conventional TLC method to microscale has a lot of advantages. In most cases, it allows to shorten time of analysis and lower the solvent consumption. Beside of that, it may also result the improvement of mixture ingredients separation, thanks to generally better efficiency of micro TLC systems in comparison with conventional TLC systems. Explanation of that fact can be easily concluded from Eq. (1) commonly used for plate number calculation in TLC [1, 2] and Van Deemter equation: The shorter distance, the lower diffusion the sharper zones. The higher the distance of migration to zone with ratio, the larger the plate number.

1 $$ N = 16\left( {\frac{{R_{F} Z_{F} }}{{W_{b} }}} \right)^{2} $$

- where:
- *N*—is plate number
- *W*_*b*_—is zone (peak) width
- *Z*_*F*_—is distance of solvent front migration
- *R*_*F*_—is retardation factor

The efficiency issue is crucial for all planar techniques, because in general it is Achilles heel of all capillary forces driven methods, which is one of the main reasons why high-performance column chromatography is being thought as method of choice for analytical investigations in present time.

Second way of reducing spot width in planar techniques is change of mobile phase grain size, which results in change of diffusion parameters in Van Deemter equation. That solution was applied in case of planar chromatography under the name of HPTLC (high-performance TLC). First papers introducing application of HPTLC dates on the middle seventies of past century [3, 4]. At present, HPTLC is often used in wide range of analyses [5–7]. The next step in that direction was switching to monolithic stationary phases. That technique was named UTLC (ultra TLC) and first papers describing it and its application dates back on 2001 [5–7]. Monolytical layers are thiner than HPTLC (10 μm). Development distance on UTLC plates is between 1 and 3 cm [8]. UTLC coupled with MS has been successfully used in analysis of peptides [9], amino acids, phenols [6] and coupled with AP MALDI-MS for analysis of other small molecules [10].

Another way of increasing planar chromatography system efficiency is inducing the mobile phase flow in the way different than capillary forces. Main disadvantage of mentioned solvent flow induction method is non-uniformity of linear mobile phase velocity down the distance of chromatogram development. In the very beginning, the velocity is higher than optimal, and down the distance of solvent front migration, it drops to values significantly lower than optimal, which leads to unnecessary band broadening in the final phase of chromatogram development.

The described above difficulty is equally present in conventional and micro TLC methods, and in order to overcome it, OPLC (overpressure layer chromatography) method was introduced [11–13], where the flow of mobile phase is induced by applying an external pressure. It proved to be an useful tool for many separations, but it also has some limitations caused mainly by non-uniform wetting of stationary phase by mobile phase down the migration distance and in the result creation of partial wettability front during chromatogram development [14]. Thus, shortening the distance of separation occurred to be useful in case of OPLC method a good action to avoid or reduce the influence of unwanted phenomenon on separation.

Next conception of separation improvement in planar techniques is connected with applying the electric field to planar chromatographic systems. First application of electric field for separation on silica gel dates back in forties of previous century [15] and is related to works on planar electrophoresis. First paper on electrochromatography was published in seventies [16] and after that the problem was left untouched for over 20 years. Further works on planar electrochromatography (ETLC) are dated on late nineties [17–20].

The technique matches advantages of electrophoresis and chromatography, and two phenomena, electrophoretic migration and electroosmotic flow, are involved in the separation improvement in this particular technique. First one is connected with electrophoretic mobility of compound molecule, and it can be calculated on the basis of Eq. (2):

2 $$ V_{ep} = \mu_{ep} E $$

- where
- *μ*_*ep*_—electrophoretic mobility of ion (substance)
- *E*—is intensity of electric field

The second one is function of field gradient and properties of solvent and stationary phase system. It can be described by Eqs. (3) and (4):

3 $$ \mu_{eo} = \left( {\frac{\varepsilon }{4\pi \eta }} \right)E\zeta $$

4 $$ \mu_{eo} = \varepsilon \varepsilon_{0} \zeta \frac{E}{\eta } $$

- where
- *μ*_*eo*_—is electroosmotic flow
- *ζ*—is zeta potential
- *ε*—is (total in Eq. 3 and relative in Eq. 4) electric permeability
- *ε*_0_—is vacuum electric permeability
- *η*—is solvent viscosity
- *E*—is intensity of electric field

The advantages of this technique are “plug like” distribution of mobile phase velocity across the sorbent bed and lack of dependence between grain size of stationary phase material and the velocity of mobile phase, and in the result short time of chromatogram development.

The biggest disadvantage of method is Joule’s heat generation, as the unwanted result of current flow in the electrochromatographic system. To reduce effect of this factor on separation, the time of chromatogram development (and, in consequence, distance of development) ought to be keep as short as possible, which is another case when the statement about the advantages of short time and distance, crucial for micro TLC method, becomes true.

## Materials and Methods

In this paper, usefulness of various planar chromatography methods such as TLC, HPTLC, OPLC UTLC and ETLC for analysis of tropane alkaloids and their derivatives separated from waste liquor after scopolamine hydrochloride crystallization of *Datura Inoxia* Mill. extract received from Wrocławskie Zakłady Zielarskie “Herbapol” was presented. Tropane alkaloids were identified on the basis of comparison with reference standards obtained from Sigma-Aldrich (St. Louis, MO, USA).

All the experiments were carried out in normal phase system, where silica gel as stationary phase was used. Chromatograms were developed on dry plates using mobile phase: methanol/acetic acid/ammonia—50/45/5 (v/v/v), chosen on the basis of available literature [21], in room temperature. Development distance of TLC experiments was 8 and 4 cm, HPTLC—4 cm, OPLC about 4 cm, UTLC and ETLC—3 cm. All used solvents were produced by Merck (Darmstadt, Germany). Horizontal TLC chambers (Chromdes, Lublin, Poland) were used for TLC, HPTLC and UTLC separations. OPLC separations were carried out using BS-50 apparatus (OPLC-NIT Budapest, Hungary). Electrochromtographic separations were performed by the use of unique ETLC system designed and produced in Planar Chromatography department, Chair of Physical Chemistry, Faculty of Chemistry, Maria Curie-Skłodowska University, Lublin, Poland.

Detection, processing and documentation of developed chromatogram except UTLC plates were performed using CAMAG (Muttenz, Switzerland) Reprostar3 equipment set and attached computer software. UTLC plates were scanned and processed using densitometer in Inorganic Chemistry Department of Medical University of Lublin.

**Fig. 1:**
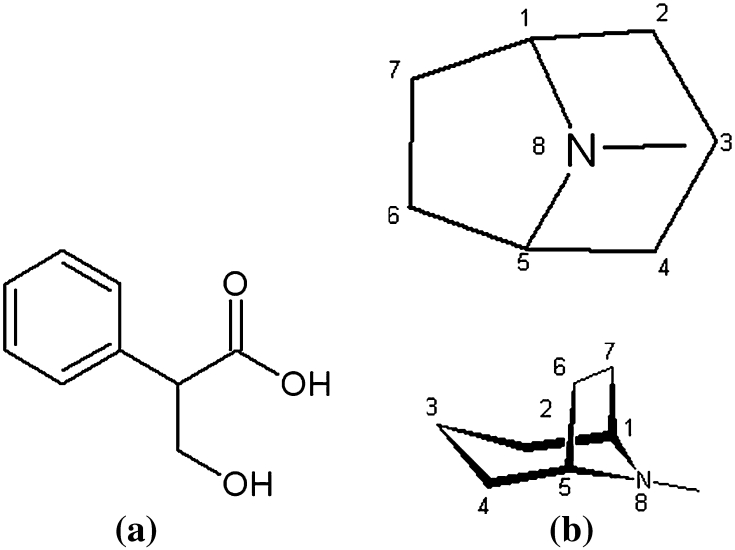
Structure of tropic acid (**a**) and tropane ring (**b**)

Tropane alkaloids and their derivatives identified in *Datura Inoxia* Mill. extract are presented in Table 1. Generally, they are a class of alkaloids, derivatives of tropic acid (Fig. 1a) and tropane ring (Fig. 1b). They occur specially in plants of Solanaceae family.

**Table 1:** Names and abbreviations used of tropane alkaloids identified in *Datura Inoxia* Mill. extract

Name	Abbreviation
Atropine sulphate	ATS
Homoatropine	HAT
l-Hyoscyamine	l-H
Scopolamine hydrobromide	SC-HBR
Tropine	TR
Scopolamine oxide	SC-N-O

Main difficulty in chromatographic analysis of this compounds in extract from *Datura inoxia* Mill. is fact that their chemical structures are similar and in some cases they differ only the one specific bound in one chiral centre.

## Results and Discussion

In first experiment as reference separation, conventional TLC technique was applied. Distance of development was 8 cm. Obtained results, such as *R*_*F*_ values, theoretical plate number separation factor and resolution are presented in Table 2.

**Table 2:**
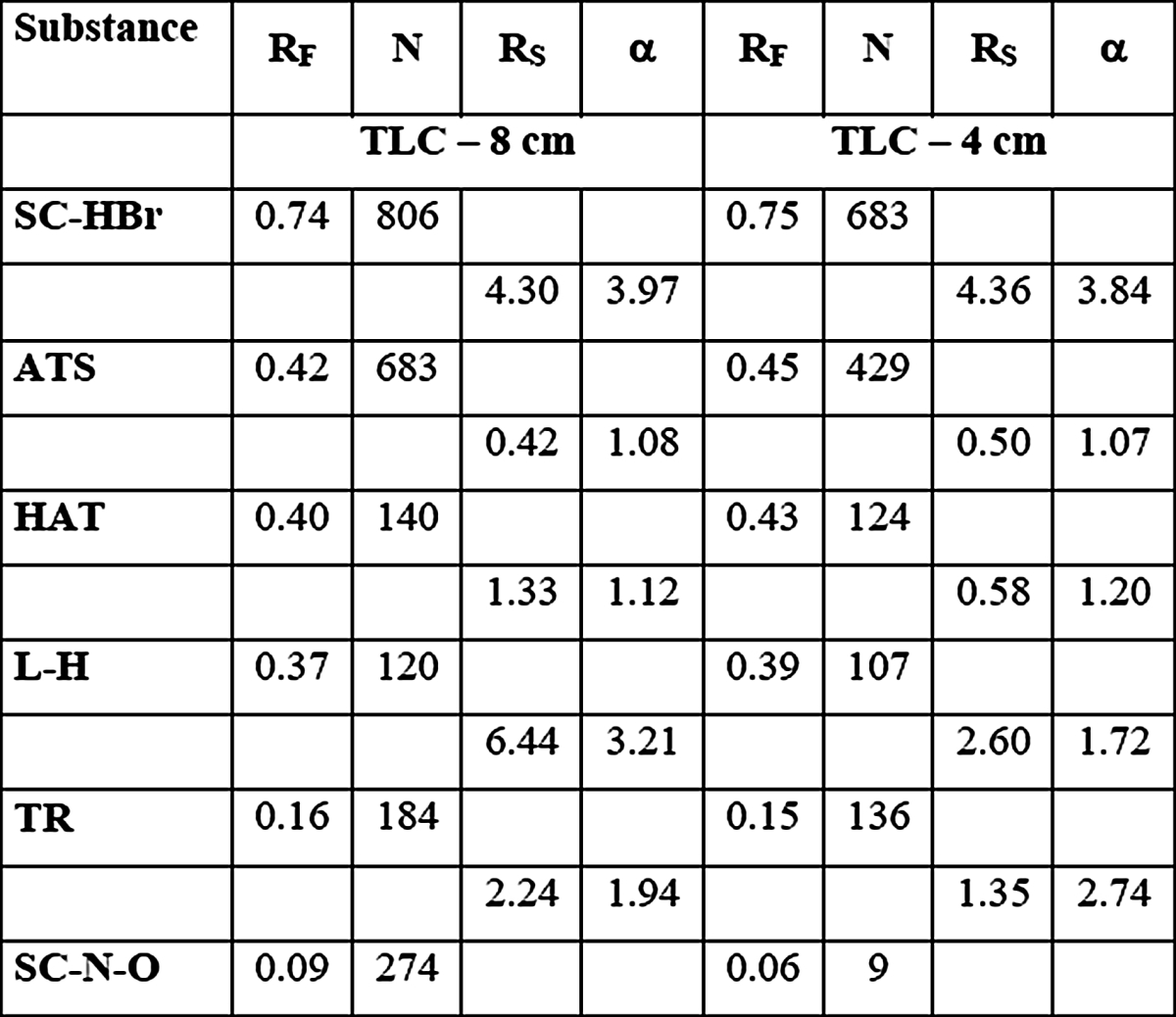
*R*_*F*_ values, plate number and separation parameters obtained for TLC chromatogram development on distance 8 and 4 cm

In the next step, distance of chromatogram development was shortened to 4 cm in order to improve time of separation and lower the solvent consumption. Intended goals were reached but shortening the distance resulted in the number of theoretical plates reduction and worsening factors of separation in case of lower migrating substances, especially between large peak atropine sulphate/homoatropine/l-hyoscyjanine and tropine peak, which was depicted in Table 2. However, spaces among the main peaks remained large enough to consider them as totally separated.

Moreover, comparing TLC results on development distance 4 cm to those obtained from TLC development on distance 8 cm, one can observe that number of theoretical plates decreased about 30 % or less, but the distance was reduces for 50 % what results in smaller height of theoretical plate. What proves that in investigated system, shortening the development distance is well grounded.

In order to improve efficiency and separation and further lowering of solvent consumption, HPTLC plates as stationary phase were used for experiment, and development distance of 4 cm was sustained.

Surprisingly, in case of HPTLC plates obtained, results were worse than on TLC system on the same distance of development. The reason of that was stationary phase overload effect, because sample amount was the same as in case of TLC development. To avoid it, initial sample was diluted [1/1 (v/v)]. Corrected results are presented in Table 3.

**Table 3:**
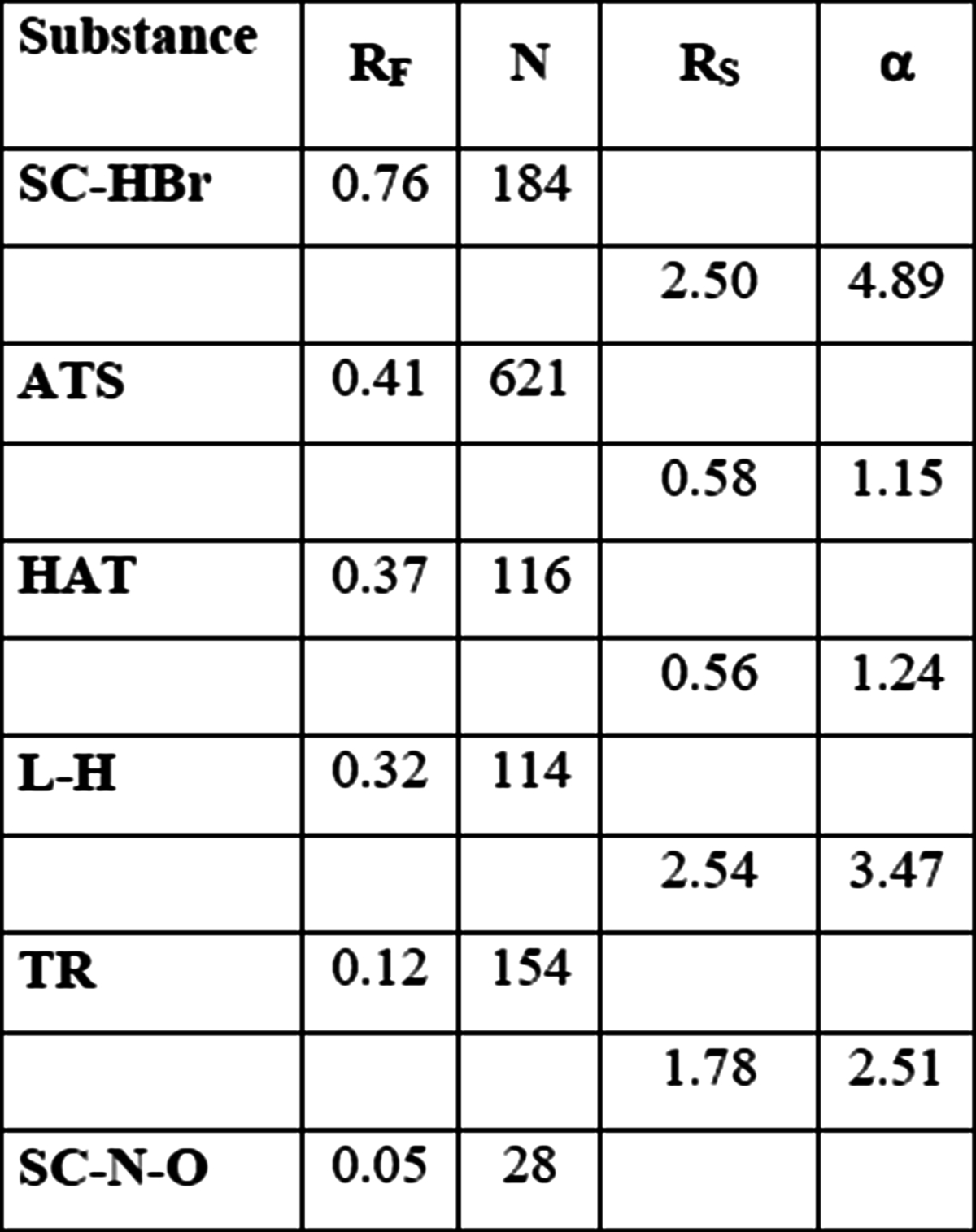
*R*_*F*_ values, plate number and separation parameters obtained for HPTLC chromatogram development on distance 4 cm

**Fig. 2:**
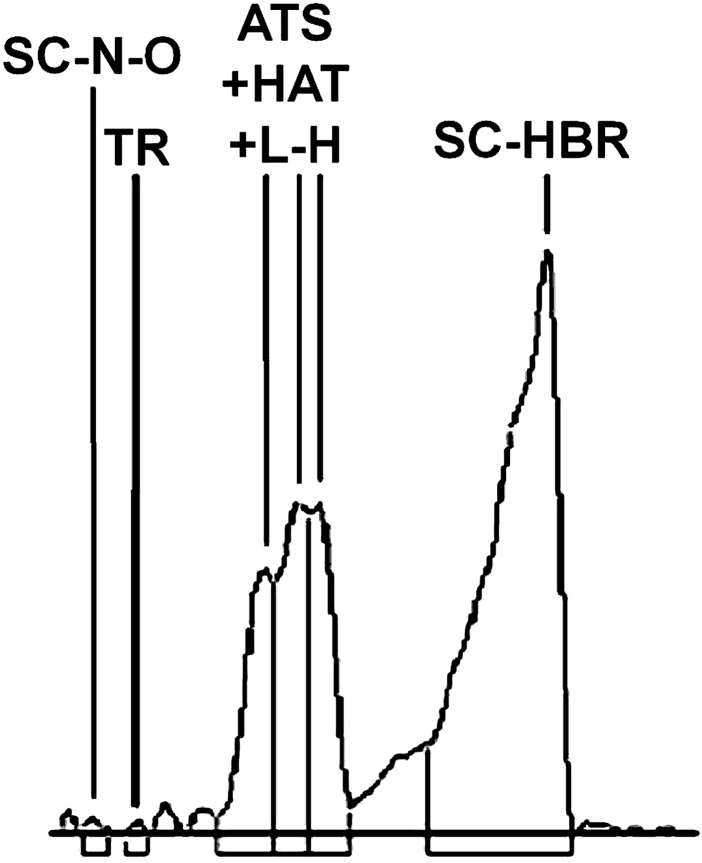
**C**hromatogram obtained for diluted extract using HPTLC plate on distance 4 cm

Even for diluted sample, in case scopolamine hydrobromide. The overload effect was visible (Fig. 2).

The consequence of overload effect is broadening of mentioned peak and drop of number of theoretical plates which in that case is lower more than three times than for TLC development on distance 4 cm. Further dilution of sample is not possible due to strong decrease of signal for other extract components below the noise. Summarizing, HPTLC technique in case of the investigated mixture appears to be worse alternative than TLC.

Although, analysis with HPTLC technique and effect of system miniaturization seemed to be quite encouraging to follow that way, and thus, the next investigated method was UTLC technique, with monolithic silica layer on the plate. In order to prevent against the overload of stationary phase, ten time smaller amount of investigated extract was applicated on plate surface. The distance of development was 3 cm. Unfortunately, results did not matched the expectations. Neither efficiency nor separation parameters were not satisfying, which was depicted in Table 4.

**Table 4:**
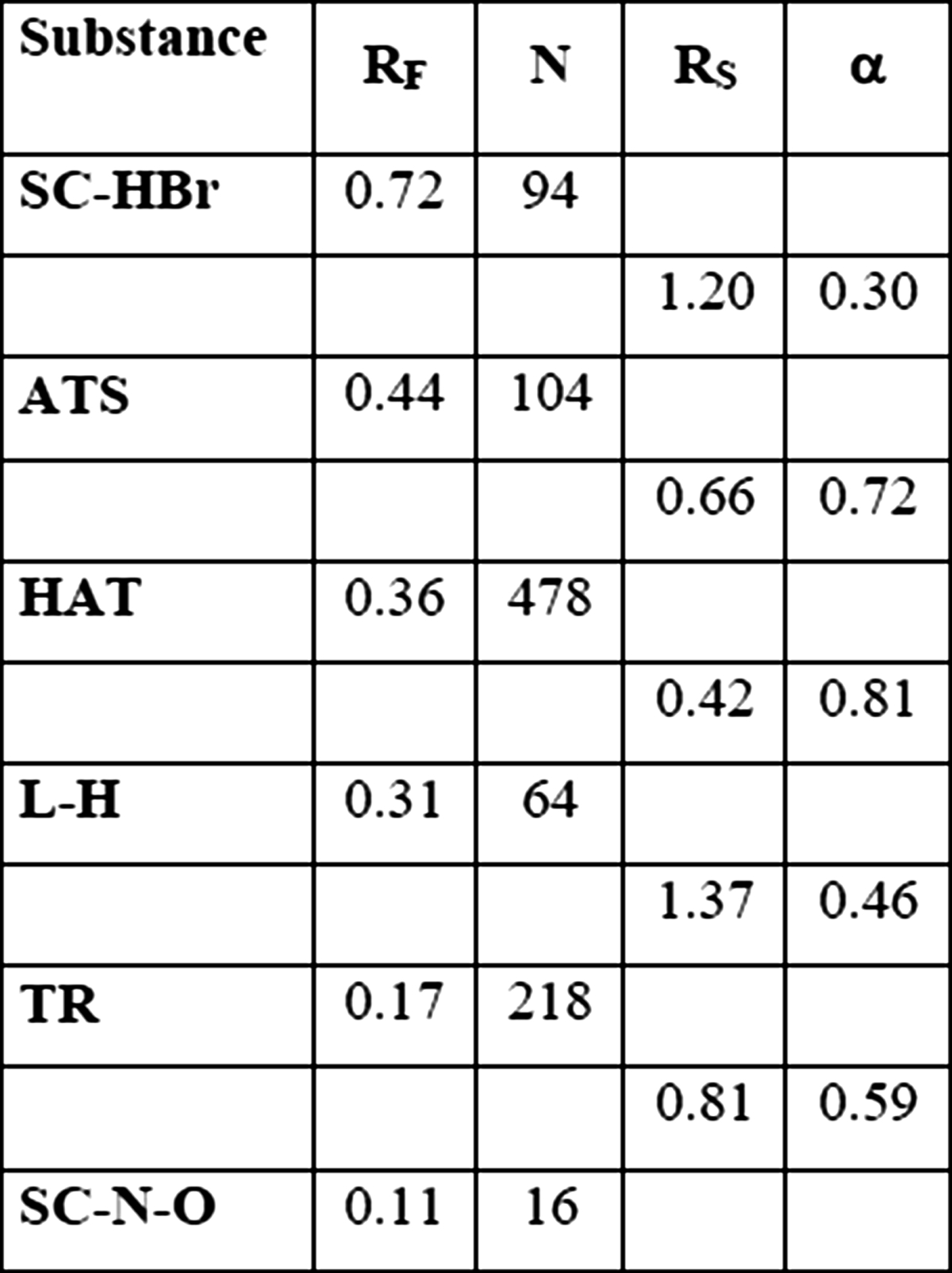
*R*_*F*_ values, plate number and separation parameters obtained for UTLC chromatogram development on distance 3 cm

In spite ten time reduction in sample amount, effect stationary phase overload in case of peak of scopolamine hydrobromide was even more visible than in HPTLC analysis. The overload effect in UTLC separation can be also observed for homoatropine peak. Practically, no efficiency or separation improvement, except tropine peak, comparing to earlier separation has been achieved. Further investigation on analysis of tropane alkaloid fraction from *Datura Inoxia* Mill. needed another method change to be continued.

All separations until this point were performed using thin-layer techniques with capillary force–induced flow and did not result in satisfying separation of extract components. Thus, decision about switching to forced flow planar techniques was made.

The first tested separation method with forced flow was OPLC (overpressure layer chromatography). TLC plates were chosen due to the fact that separation obtained on HPTLC plates in this technique was not particularly better than in case of standard TLC plates. Method parameters such as rapid flush volume, volume and velocity of mobile phase were previously optimized in earlier studies. OPLC separation results are presented in Table 5.

**Table 5:**
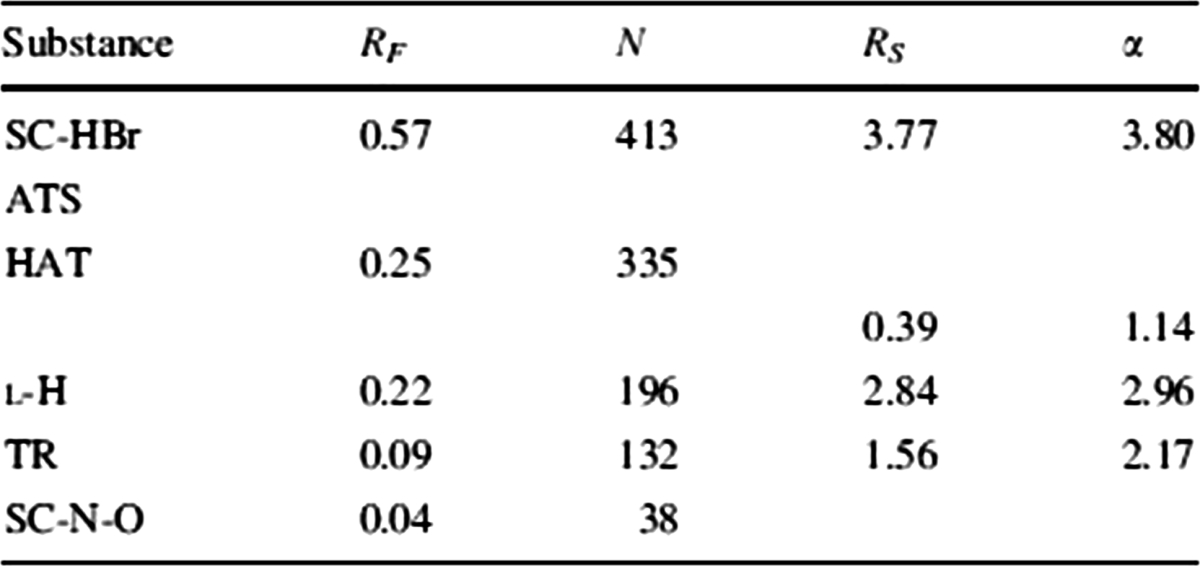
*R*_*F*_ values, plate number and separation parameters obtained for OPLC chromatogram development on distance 4 cm

Applying this technique did not bring the solution. Significant efficiency improvement was observed only in case of scopolamine oxide. ATS/HAT/l-H separation was worse than in case of earlier investigated TLC/HPTLC techniques.

Since obtained results were still unsatisfying, it was decided to apply another forced flow thin-layer technique—ETLC (electro TLC), also known as PEC (planar electrochromatography).

**Fig. 3:**
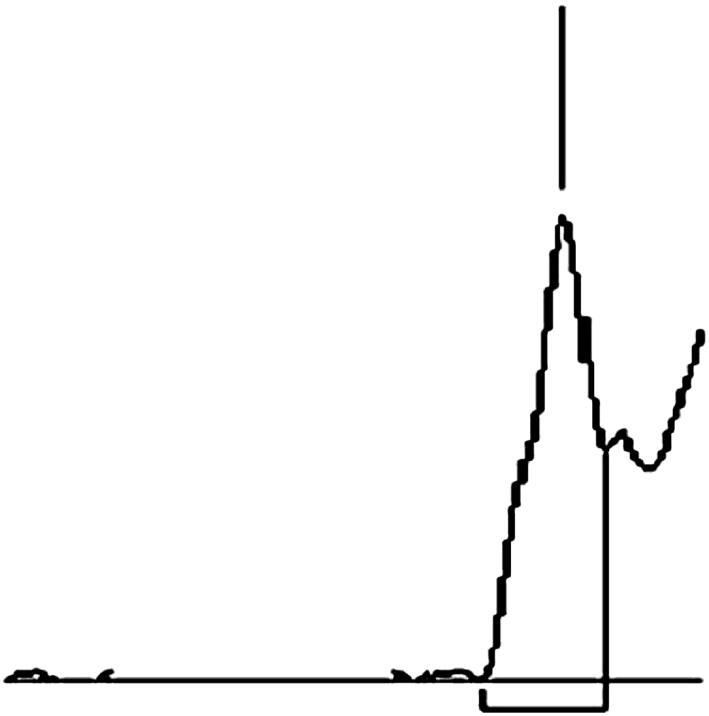
Chromatogram obtained after development, using ETLC technique on cathode side. Distance of chromatogram development—3 cm intensity of field 0.17 kV cm^−1^

Separations were carried out on dry plates and on open sorbent bed on TLC plates. Developments on cathode and anode sides were carried out simultaneously on distance of 3 cm. Initial field gradient was 0.17 kV cm^−1^. Chromatograms obtained on both sides were totally different. On cathode side, after chromatogram development components remained unseparated. On anode side, two bands were detected Scopolamine hydrobromide and one big band for five other separated tropane alkaloids. On cathode side, no separation was observed (Fig. 3).

**Fig. 4:**
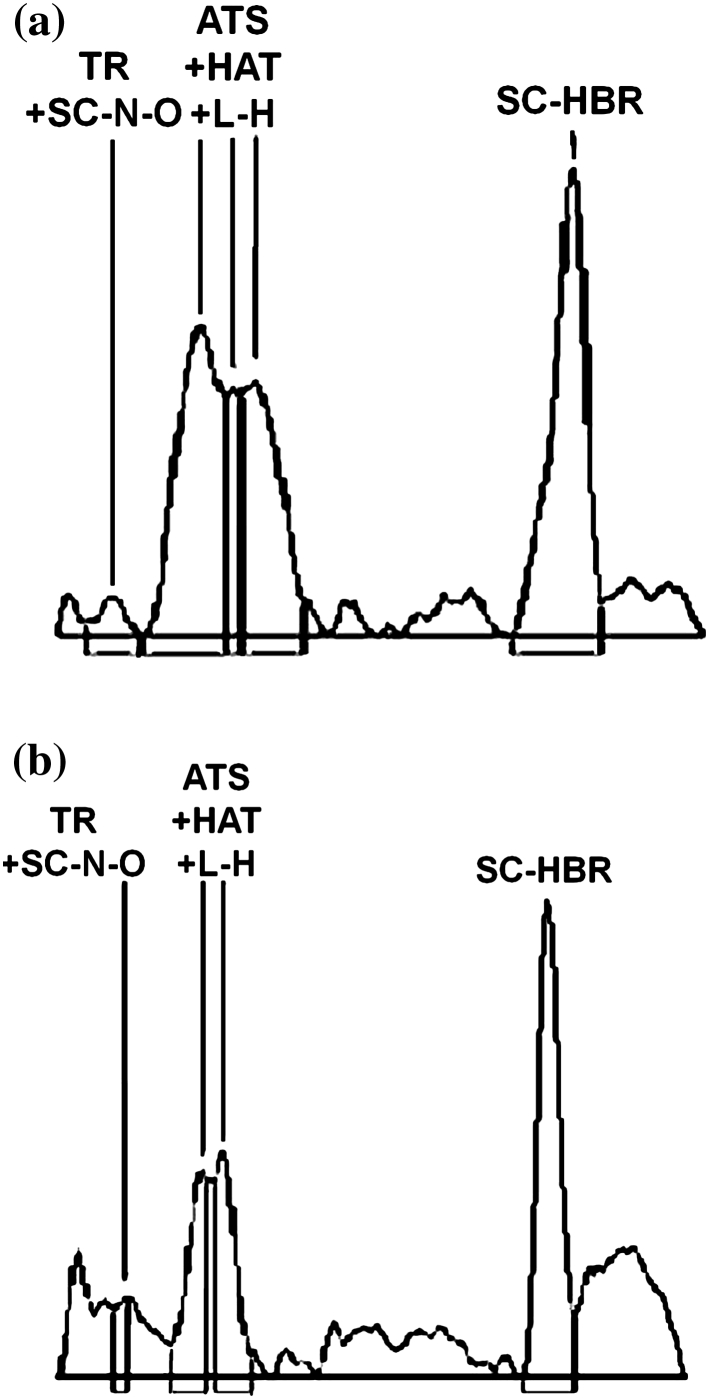
Chromatograms obtained after separation, using ETLC technique on cathode side (**a**) and anode side (**b**). Stationary phase: Silica gel 60 F254 TLC plates. Distance of chromatogram development 4 cm intensity of field 0.5 kV cm^−1^

Increasing the intensity of applied electric field until the gradient reached 0.5 kV cm^−1^ finally resulted in separation improvement inside ATS/HAT/l-H peak. Similarly, as it was shown on the previously described chromatogram development, cathode side chromatogram differs from anode side chromatogram what was presented in Table 6 and on Fig. 3a, b. On cathode side, significant plate number increase has been noticed as a result of intensity of electric field growth (Fig. 4).

**Table 6:**
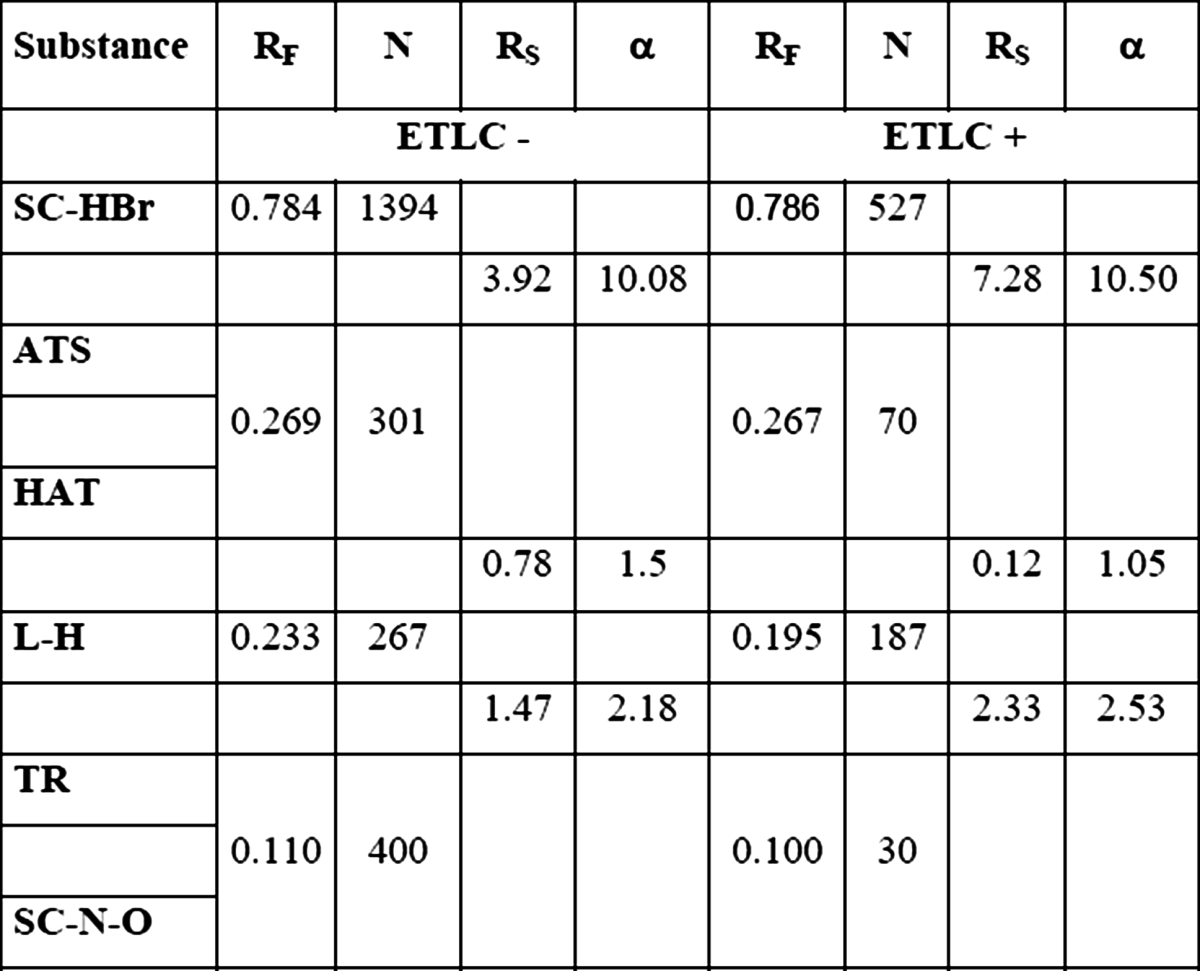
*R*_*F*_ values, plate number and separations parameters obtained for ETLC chromatogram development on cathode side (ETLC−) and anode side (ETLC+)

Distance of development 3 cm

## Conclusions

Applying the micro TLC techniques in investigation on analysis of tropane alkaloids from *Datura Inoxia* Mill. extract had more positive than negative effects. Though that efficiency of chromatographic system was lowered in case of capillary force–induced flow methods, it was still enough to separate the peaks and obtain even better separation inside atropine sulphate/homoatropine/l-hyoscyamine peak.

Employing planar electrochromatography technique for the extract analysis resulted not only in further shortening the distance and time of chromatogram development. ETLC technique occurred also the most efficient separation method comparing to all techniques taken under the investigation in given conditions. Electro thin-layer chromatography proved to be an alternative separation method for components of plant extracts. Further investigation on that method should be directed to improve Joule’s heat transfer from the apparatus and separating sorbent bed from gaseous phase over it what allows to reach this technique its full separation potential.
